# Immune cell signatures and preeclampsia: Unveiling causal links through genome-wide association studies

**DOI:** 10.1097/MD.0000000000046002

**Published:** 2026-01-09

**Authors:** Huale Zhang, Yizheng Zu, Xiaoyan Xiu, Huangchang Yi, Xia Xu, Jianying Yan

**Affiliations:** a Fujian Maternity and Child Health Hospital College of Clinical Medicine for Obstetrics & Gynecology and Pediatrics, Fujian Medical University, Fuzhou, China; b Fujian Clinical Research Center for Maternal-Fetal Medicine, Fuzhou, China; c Laboratory of Maternal-Fetal Medicine, Fujian Maternity and Child Health Hospital, Fuzhou, China; d National Key Obstetric Clinical Specialty Construction Institution of China, Fuzhou, China; e The Second People's Hospital of Changzhou, the Third Affiliated Hospital of Nanjing Medical University, Changzhou, China.

**Keywords:** causal relationship, immunity, MR analysis, placenta, preeclampsia

## Abstract

The immune-placental interaction could potentially contribute both causally and therapeutically to preeclampsia (PE). However, previous observational studies have not demonstrated a causal association between these 2 factors. Exploring the causal relationship between immune cells and PE provides genetic evidence that immune cells play a role in PE risk. Using a large dataset of genome-wide association studies, we conducted a bidirectional Mendelian randomization (MR) analysis to determine whether 731 immune cell signatures were correlated with PE, which included 4 types of immune signatures. After false discovery rate (FDR) correction, 3 immunophenotypes were identified to be significantly associated with PE risk: human leukocyte antigen on dendritic cells (odds ratio [OR] = 1.072, 95% confidence interval [CI] = 1.0352–1.110, *P*_FDR_ = .012), absolute T cell count (OR = 1.477, 95% CI = 1.281–1.703, *P*_FDR_ = 1.71 × 10^−5^), and absolute lymphocyte count (OR = 1.514, 95% CI = 1.306–1.755, *P*_FDR_ = 1.58 × 10^−5^). Furthermore, PE did not affect immunophenotypes significantly after FDR adjustment, but 12 suggestive immunophenotypes were detected without FDR adjustment, including CD3 on activated and secreting CD4 regulatory T cell (*β* = 0.467, *P* = .039), CD3 on CD39 + CD4 + T cell (*β* = 0.508, *P* = .024), CD3 on CD45RA + CD4 + T cell (*β* = 0.515, *P* = .022), CD3 on CD28 + CD45RA + CD8 + T cell (*β* = 0.494, *P* = .028), CD3 on CD39 + CD8 + T cell (*β* = 0.486, *P* = .029), CD45 on HLA DR + CD8 + T cell (*β* = 0.518, *P* = .018), CD3 on secreting CD4 regulatory T cell (*β* = 0.508, *P* = .025), CD25 on resting CD4 regulatory T cell (*β* = 0.496, *P* = .018), CD4 on CD39 + activated CD4 regulatory T cell (*β* = 0.445, *P* = .048), CD3 on CD28 + CD45RA − CD8 + T cell (*β* = 0.498, *P* = .024), CD3 on effector memory CD4 + T cell (*β* = 0.614, *P* = .007), and CD3 on activated CD4 regulatory T cell(*β* = 0.444, *P* = .049). Using genetic methods, this study uncovers the close relationship between immune cells and PE, which provides guidance for future clinical trials.

## 1. Introduction

Preeclampsia (PE) is defined as new-onset hypertension after 20 weeks of gestation accompanied by proteinuria or other multiple organ dysfunction. Severe cases may be accompanied by lesions in multiple organ lesions.^[1–3]^ PE is a pregnancy-specific complication and the second leading cause of direct maternal death, with an estimated prevalence of 2% to 8% worldwide,^[4]^ resulting in an estimated 70,000 maternal deaths and 500,000 fetal and neonatal deaths annually.^[5]^ The risk of placental abruption, premature delivery, acute renal failure, and cardiovascular and cerebrovascular diseases in pregnant women with PE is significantly increased, as well as intrauterine hypoxia, growth restriction, and even fetal death, which seriously endangers the safety of the mother and child and places a heavy health and economic burden on society, families, and individuals.^[6–9]^ At present, the main treatment methods are only symptomatic and supportive treatments such as lowering blood pressure and spasmolysis, which cannot effectively improve maternal and fetal outcomes.

Key mechanisms underlying the development of hypertensive disorders of pregnancy include endothelial dysfunction, angiogenesis, impaired spiral uterine artery remodeling, and insufficient trophoblast infiltration,^[10,11]^ but in recent years, the maternal immune system has been emphasized to play a key role in the development of PE.^[12,13]^ The normal growth and development of the fetus must rely on maternal immune tolerance, and an imbalance in maternal immune tolerance and the subsequent inflammatory response leads to pathological pregnancy, including PE. Immune cells are dynamic during pregnancy, and spiral artery remodeling in early pregnancy is dependent on the anti-inflammatory environment to ensure maternal–fetal tolerance.^[12,14]^ However, in contrast to normal pregnancy (NP), pro-inflammatory cytokines such as TNF, IL-6, and IL-17 are elevated during PE and promote cytotoxic inflammatory responses.^[15–17]^ Similarly, in NP, macrophages favor M2, whereas in PE, the balance shifts toward the M1 phenotype, and M1 cells secrete soluble FMS-like tyrosine kinase-1, which is associated with a proinflammatory response.^[12]^ In PE, B-1a cells are stimulated to produce an angiotensin II type 1 receptor agonist autoantibodies, which induces signaling pathways leading to vasoconstriction and aldosterone secretion, thereby stimulating the renin-angiotensin system and increasing blood pressure. In conclusion, immune dysregulation and inflammation are important factors in placental dysfunction, which ultimately lead to elevated maternal blood pressure and end-organ damage. Despite extensive research, the results regarding the association between immune inflammation and PE are inconsistent.

Mendelian randomization (MR) is a method that uses genetic variants as instrumental variables (IVs) to infer the causality between exposure factors and outcomes in observational studies. Previous observational studies have found many associations between immune cells and PE, supporting the hypothesis of an association between the two. In this study, comprehensive two-sample MR analysis was used to determine the causal relationship between immune cells and PE. To provide a relevant theoretical basis for the relationship between immune cells and the occurrence and development of PE.

## 2. Materials and methods

### 2.1. Study design

We assessed the causal relationship between 731 immune cell signatures (7 groups) and PE based on bidirectional two-sample MR analysis. MR uses genetic variation to represent risk factors; therefore, valid IVs in causal inference must satisfy 3 key assumptions: genetic variation is directly associated with exposure; genetic variation is not associated with possible confounders between exposure and outcome; and genetic variation does not affect outcome through pathways other than exposure. The studies included in our analysis were approved by the relevant institutional review boards and the participants provided informed consent.

### 2.2. Genome-wide association study (GWAS) data sources for PE

PE heritability is estimated to be 50% to 55%, with a maternal genetic contribution risk of 30% to 35% and a fetal genetic contribution risk of 20%.^[18]^ The genetic association of PE was extracted from FinnGen consortium data. The FinnGen consortium performed a large GWAS to identify genetic variants associated with PE in 9, 120 patients and 293,373 controls of Finnish ancestry (https://risteys.finregistry.fi/endpoints/O15_PRE_OR_ECLAMPSIA).

### 2.3. Immunity-wide GWAS data sources

GWAS summary statistics for each immune trait are publicly available from the GWAS Catalog (accession numbers GCST90001391 to GCST90002121).^[19]^ A total of 731 immunophenotypes were included, including absolute cell (AC) counts (n = 118), median fluorescence intensities (MFI) reflecting surface antigen levels (n = 389), morphological parameters (MP; n = 32), and relative cell (RC) counts (n = 192). Specifically, the MFI, AC, and RC features contained B cells, CDCs, mature T cells, monocytes, myeloid cells, TBNK (T cells, B cells, and natural killer cells), and Treg panels, whereas the MP feature contained CDC and TBNK panels. The original GWAS on immune traits was performed using data from 3757 European individuals, and there were no overlapping cohorts. Approximately 22 million single nucleotide polymorphisms genotyped with high-density arrays were imputed using the Sardinian sequence-based reference panel, and associations were tested after adjusting for covariates (i.e., sex, age, and age^2^).

### 2.4. Selection of IVs

In accordance with recent research,^[19,20]^ the significance level of the IVs for each immune trait was set to 1 × 10^−5^. The clumping procedure in PLINK software (version v1.90) was used to prune these single nucleotide polymorphisms (linkage disequilibrium *r*^2^ threshold < 0.1 within 500 kb distance), where linkage disequilibrium *r*^2^ was calculated based on 1000 genomes projects as a reference panel. For PE, we adjusted the significance level to 5 × 10^−8^. The proportion of phenotypic variation explained and the *F* statistic were calculated for each IV to evaluate IV strength and avoid weak instrumental bias. A total of 7 to 1786 independent IVs for immunophenotypes were determined, and these generated IVs explained an average of 0.240% (range 0.004–3.652%) of the variance in their respective immune traits. After removing the IVs with low *F*-statistics (< 10), 108 IVs for PE were preserved for further analysis.

### 2.5. Statistical analysis

Analyses were conducted using R 4.3.2 (http://www.r-project.org). Based on the “Mendelian-Randomization” package (version 0.4.3), the inverse variance weighting (IVW), weighted median, weighted modes, and MR-Egger methods were used to assess causal associations between 731 immunophenotypes and PE. In order to test the heterogeneity among selected IVs, Cochran *Q* statistic was applied and corresponding *P* values were calculated. As an alternative to fixed-effects IVW, random effects IVW was used if the null hypothesis was rejected. We excluded horizontal pleiotropy by using MR-Egger, which implies horizontal multiplicity if the intercept term is significant. Furthermore, we also analyzed the results using scatter plots and funnel plots. Scatter plots revealed no outliers.

## 3. Results

### 3.1. Exploration of the causal effect of immunophenotypes on PE

**Figure 1.**
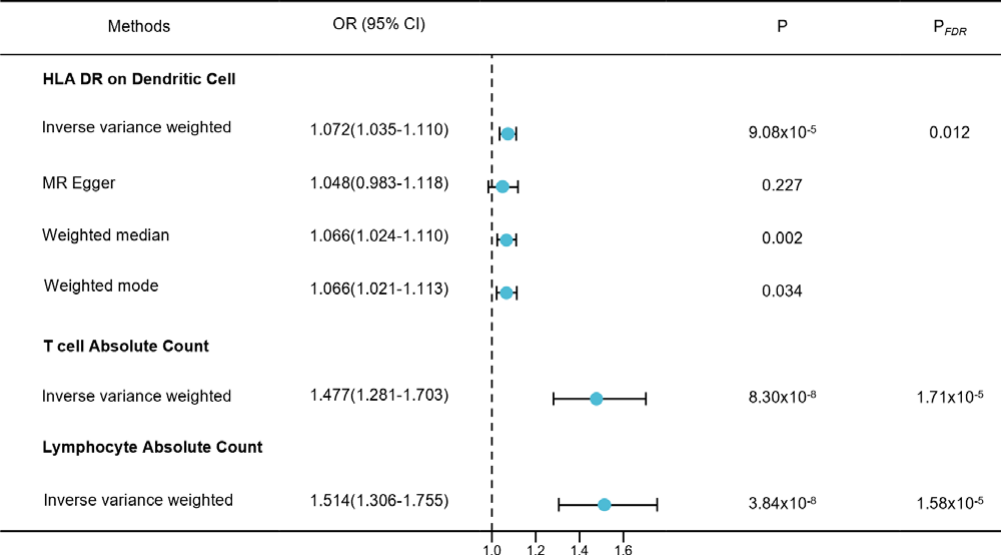
Forest plots showed the causal associations between immune cell traits and PE by using different methods. Forest plots showing the causal estimates of 3 immune cell traits that are significantly associated with PE in multiple test adjustments with inverse-variance weighted, MR Egger, weighted median and weighted mode. CI = confidence interval, HLA-DR = human leukocyte antigen, OR = odds ratio, MR = Mendelian randomization, PE = preeclampsia.

We mainly utilized the IVW method to analyze the two-sample MR data to examine the causal effects of immunophenotypes on PE. Based on the false discovery rate (FDR) method (*P*_FDR_ < .05), multiple test adjustments revealed the pathogenicity of PE in 3 immunophenotypes: human leukocyte antigen (HLA-DR) in dendritic cells (cDC panel), T cell absolute count (TBNK panel), and lymphocyte absolute count (TBNK panel). Specifically, the odds ratio (OR) of HLA DR on dendritic cells (DCs) for PE risk was estimated to be 1.072 (95% confidence interval [CI] = 1.0352–1.110, *P* = 9.08 × 10^−5^, *P*_FDR_ = .012, Fig. 1, Table S1, Supplemental Digital Content, https://links.lww.com/MD/Q821) using the IVW method. Similar results were observed using the 3 other methods: MR-Egger (OR = 1.048, 95% CI = 0.983–1.118, *P* = .227), weighted median (OR = 1.066, 95% CI = 1.024–1.110, *P* = .002), and weighted mode (OR = 1.066, 95% CI = 1.021–1.113, *P* = .034). The OR of absolute T cell count on PE risk was estimated to be 1.477 (95% CI = 1.281–1.703, *P* = 8.30 × 10^−8^, *P*_FDR_ = 1.71 × 10^−5^, Fig. 1, Table S1, Supplemental Digital Content, https://links.lww.com/MD/Q821) using the IVW method. The OR of absolute lymphocyte count on PE risk was estimated to be 1.514 (95% CI = 1.306–1.755, *P* = 3.84 × 10^−8^, *P*_FDR_ = 1.58 × 10^−5^, Fig. 1, Table S1, Supplemental Digital Content, https://links.lww.com/MD/Q821) using the IVW method. Based on the results of the other 3 methods and sensitivity analysis, the findings of the causal association were robust. Specifically, the result of Cochran *Q* test ruled out the possibility of heterogeneity, and the intercept of MR-Egger ruled out the possibility of horizontal pleiotropy (Tables S2 and S3; Fig. S1, Supplemental Digital Content, https://links.lww.com/MD/Q820). The results were also shown to be stable using scatter, funnel, and leave-one-out plots (Fig. S1, supplemental Digital Content, https://links.lww.com/MD/Q820).

### 3.2. Exploration of the causal effect of PE onset on immunophenotypes

**Figure 2.**
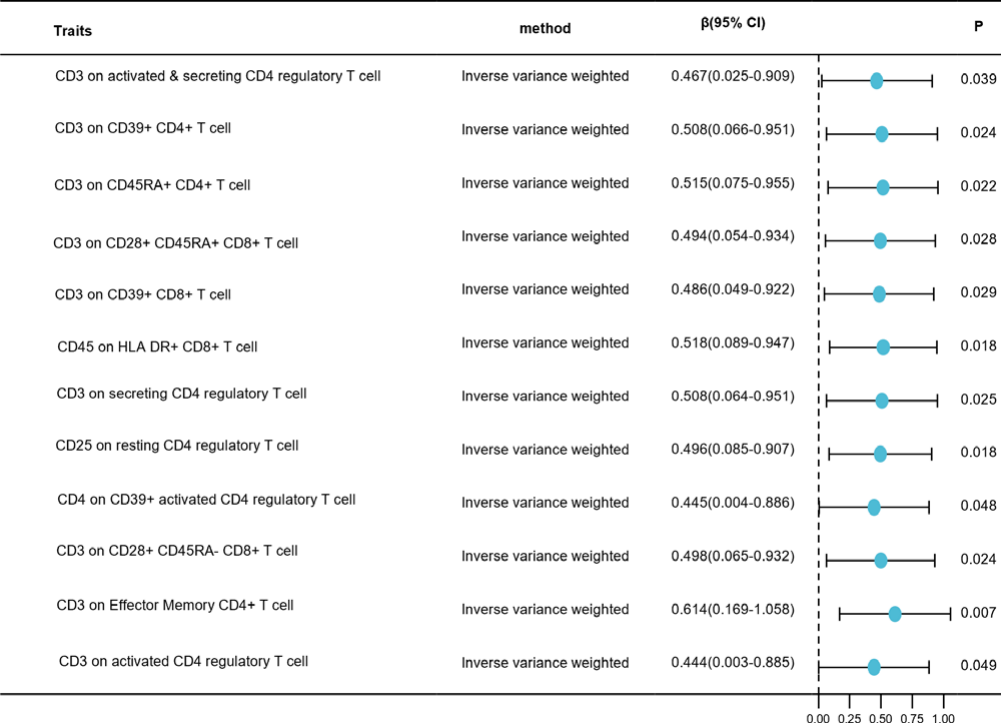
Forest plots showed the causal associations between PE and immune cell traits by using IVW method. Forest plots showing the causal estimates of PE that are associated with 12 immunophenotypes by using IVW method without FDR adjustment. CI = confidence interval, FDR = false discovery rate, IVW = inverse-variance weighted, PE = preeclampsia.

Two-sample MR analysis using the IVW method as the main analysis method was used to investigate the causal effects of PE on immunophenotypes. No immune traits were identified after FDR adjustment. Twelve suggestive immunophenotypes were detected without FDR adjustment, of which 10 belonged to the Treg panel, one to the maturation stages of the T cell panel, and one to the TBNK panel (Table S4, Supplemental Digital Content, https://links.lww.com/MD/Q821). According to our findings, PE onset increased the level of CD3 in activated and secreted CD4 regulatory T cells (*β* = 0.467, 95% CI = 0.025–0.909, *P* = .039). A significant increase in CD3 expression in CD39 + CD4 + T cells was observed in PE patients (*β* = 0.508, 95% CI = 0.066–0.951, *P* = .024). It was also found that CD3 expression on CD45RA + CD4 + T cells was increased in patients with PE (*β* = 0.515, 95% CI = 0.075–0.955, *P* = .022). There was also an increase in CD3 on CD28 + CD45RA + CD8 + T cells in patients with PE (*β* = 0.494, 95% CI = 0.054–0.934, *P* = .028). It was also found that CD3 expression on CD39 + CD8 + T cells was increased in patients with PE (*β* = 0.486, 95% CI = 0.049–0.922, *P* = .029). A significant increase in CD45 expression in HLA-DR + CD8 + T cells was observed in PE patients (*β* = 0.518, 95% CI = 0.089–0.947, *P* = .018). CD3 secretion by CD4 regulatory T cells also increased in patients with PE (*β* = 0.508, 95% CI = 0.064–0.951, *P* = .025). There was also an increase in CD25 expression in resting CD4 + regulatory T cells in PE patients (*β* = 0.496, 95% CI = 0.085–0.907, *P* = .018). CD4 on CD39 + activated CD4 regulatory T cells also increased in patients with PE (*β* = 0.445, 95% CI = 0.004–0.886, *P* = .048). A significant increase in CD3 on CD28 + CD45RA − CD8 + T cells was observed among patients with PE (*β* = 0.498, 95% CI = 0.065–0.932, *P* = .024). There was also an increase in CD3 expression in effector memory CD4 + T cells in PE patients (*β* = 0.614, 95% CI = 0.169–1.058, *P* = .007). CD3 expression on activated CD4 regulatory T cells was also increased in patients with PE (*β* = 0.444, 95% CI = 0.003–0.885, *P* = .049; Fig. 2). It was evident from the sensitivity analysis that the causal associations observed were robust (Figs. S2–S4, supplemental Digital Content, https://links.lww.com/MD/Q820). Cochran *Q* test excluded heterogeneity (Table S5, Supplemental Digital Content, https://links.lww.com/MD/Q821).

## 4. Discussion

In this study, we investigated the causal associations between 731 immune cell traits and PE, using large publicly available genetic datasets. To our knowledge, this is the first MR analysis to explore the causal relationship between the multiple immunophenotypes of PE. In this study, among the 4 types of immune traits (MFI, RC, AC, and MP), 3 immunophenotypes had significant causal effects on PE (*P*_FDR_ < .05), and PE was found to have causal effects on 12 immunophenotypes (Without FDR adjustment).

Our study found that the risk of PE increased with the proportion of HLA-DR in DCs. DCs are the most powerful antigen-presenting cells that exist at the maternal–fetal interface, circulate in the endometrium and decidua, and play a key role in inducing the activation and differentiation of native T-cells into Th1, Th2, Th17, and Treg.^[21]^ Changes in the absolute number of DCs and distribution of DC subtypes are necessary for the maintenance of pregnancy.^[22]^ Evidence suggests that DCs play a role in the development and progression of PE. Compared with healthy pregnant women, the expression of myeloid DC cells in the peripheral blood of the PE and eclampsia groups was higher than that of the control group.^[23]^ HLA-DR is an MHC class II cell surface receptor encoded by the HLA-DR complex on chromosome 6 of 6P21. A recent study suggested the expression of HLA-DR by syncytiotrophoblasts in placentae from pregnancies complicated with PE, potentially forming an immunogenic trigger for the maternal immune system.^[24]^ However, detailed functional analysis of HLA-DR in DC-mediated immunity in PE species remains to be performed.

NP is similar to a successful semi-allograft transplantation. The establishment and maintenance of NP depend on the formation of maternal–fetal immune tolerance, which involves a variety of immune cells, especially T cells.^[21]^ Activated maternal CD4 + T cells can differentiate into different subsets to regulate proinflammatory and anti-inflammatory signals and release cytokines to initiate different maternal and fetal immune responses.^[25]^ Previous studies have demonstrated a correlation between PE and cytokine secretions. Compared to healthy pregnant women, proinflammatory cytokines (TNF-α, IFN-γ, IL-2, IL-8, and IL-6) are significantly increased in the peripheral blood of PE patients.^[26]^ Furthermore, our research revealed that an increase in the T cell absolute count ratio is associated with an elevated risk of PE rather than PE, causing abnormalities in absolute T cell values.

Absolute lymphocyte count is the number of lymphocytes in the white blood cell count, which is a specific immunocompetent cell. It is essential for the immune system to perform immune surveillance and maintain internal environmental balance.^[27,28]^ Studies have shown increased white blood cell counts in patients with PE, especially in those with severe PE.^[29]^ At the same time, some scholars have proposed that PE neutrophils and lymphocytes can release a variety of inflammatory cytokines to activate inflammatory cells and immune responses, leading to endothelial dysfunction.^[30]^ Interestingly, our research found that absolute lymphocyte count, rather than PE, is the cause of the increased incidence of PE, leading to an abnormal absolute lymphocyte count. These findings may serve as a reference for future biological research.

In addition, it is noteworthy that the presence of PE was associated with increased CD3 on activated and secreting CD4 regulatory T cells, CD3 on secreting CD4 regulatory T cells, CD3 on activated CD4 regulatory T cells, CD4 on CD39 + activated CD4 regulatory T cells, CD25 on resting CD4 regulatory T cells, CD3 on CD39 + CD4 + T cells, CD3 on CD45RA + CD4 + T cells, CD3 on CD28 + CD45RA + CD8 + T cells, CD3 on CD39 + CD8 + T cells, CD45 on HLA-DR + CD8 + T cells, CD3 on CD28 + CD45RA − CD8 + T cells, and CD3 on effector memory CD4 + T cells. Regulatory T cells (Tregs) are a subset of T cells that control autoimmune reactivity and are closely related to the body’s pro-inflammatory response and autoimmune diseases. Their functions are directly related to HLA-DRs and they can secrete a variety of inflammatory factors. There is evidence that T cell subsets are involved in the pathogenesis and progression of PE, and compared with normal healthy pregnant women, PE pregnant women have reduced Treg cell number and function, while Th1 and Th17 cell subsets are increased, and this imbalance between subsets of cells may be one of the causes of inflammation.^[31,32]^ However, there is also evidence of an increased percentage of CD4 + CD25^high+^ + FoxP3^+^ Treg cells in the peripheral blood circulation in PE pregnant women compared to that in healthy pregnant women,^[33]^ which is consistent with our findings of an increased number of Treg cells in PE patients. These increased Treg cells represent a distinct Treg cell population that may be induced from peripheral native T cells by fetal alloantigens, and the increased allot cell response may be associated with PE development.^[34]^

This study conducted a two-sample MR analysis based on published large GWAS cohort results, with a large sample size of approximately 150,000 people and a high statistical efficiency. The conclusions of this study are based on genetic IVs and the use of the MR analysis method for causal reasoning. The results were robust and were unaffected by horizontal pleiotropy or other factors. However, our study had some limitations. First, horizontal pleiotropy cannot be fully evaluated even when multiple sensitivity analyses are conducted. Second, due to the lack of individual information, we were unable to conduct further stratified analyses of the population. Finally, the study was based on a European database; therefore, the conclusion cannot be extended to other racial groups, which limits the universality of our results.

## 5. Conclusion

In summary, our comprehensive bidirectional Mendelian studies have demonstrated a causal relationship between several immunophenotypes and PE, highlighting the complex interaction between the immune system and PE, which may provide new approaches for the immunological mechanism and early intervention of preeclampsia, and also provide valuable clues for the future clinical prevention of preeclampsia.

## Acknowledgments

We thank all authors for their contributions.

## Author contributions

**Data curation:** Huangchang Yi.

**Formal analysis:** Huale Zhang, Huangchang Yi, Xia Xu.

**Funding acquisition:** Huale Zhang, Jianying Yan.

**Methodology:** Xiaoyan Xiu, Xia Xu.

**Software:** Huale Zhang.

**Validation:** Xiaoyan Xiu.

**Writing – original draft:** Huale Zhang, Yizheng Zu, Xiaoyan Xiu, Xia Xu.

**Writing – review & editing:** Yizheng Zu, Jianying Yan.

## Supplementary Material




