# Supplementary material for: Immune cell signatures and preeclampsia: Unveiling causal links through genome-wide association studies
Source: Medicine (Baltimore). 2026 Jan 9;105(2):e46002. doi: 10.1097/MD.0000000000046002 (PMC12795097; doi:10.1097/MD.0000000000046002)

**Fig.S1 Causal effects of immunophenotypes on PE.** A: Funnel plot between Lymphocyte Absolute Count and PE risk; B: Funnel plot between T cell Absolute Count and PE risk; C: Funnel plot between HLA DR on Dendritic Cell and PE risk; D: Scatter plot between Lymphocyte Absolute Count and PE risk; E: Scatter plot between T cell Absolute Count and PE risk; F: Scatter plot between HLA DR on Dendritic Cell and PE risk; G: Forest plot between Lymphocyte Absolute Count and PE risk; H: Forest plot between T cell Absolute Count and PE risk; I: Forest plot between HLA DR on Dendritic Cell and PE risk; J: Leave-One-Out plot HLA DR on Dendritic Cell and PE risk.

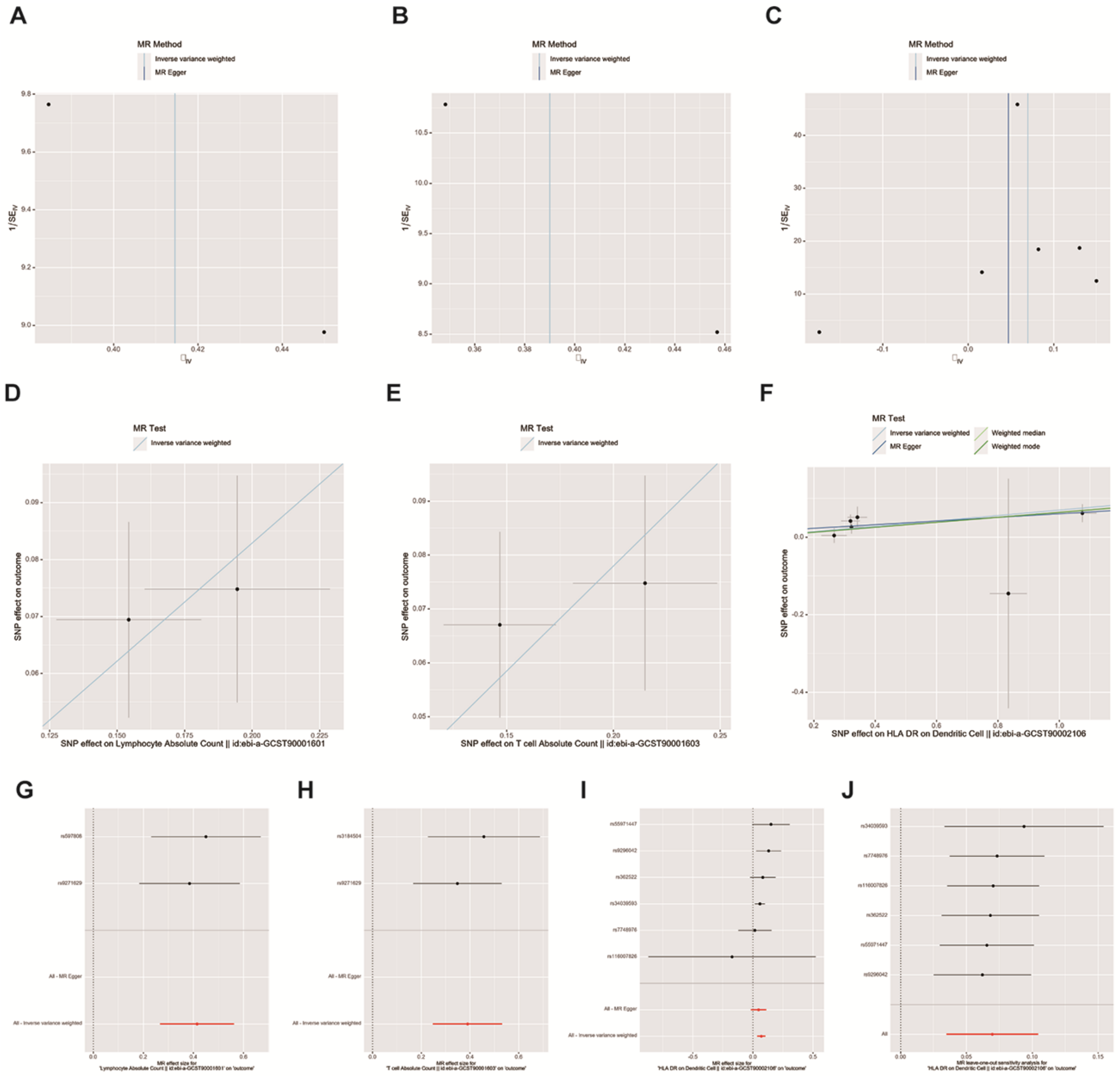

**Fig.S2 Funnel plot between PE and immune cells.** A: Funnel plot between CD3 on Effector Memory CD4+ T cell and PE risk; B: Funnel plot between CD3 on activated CD4 regulatory T cell and PE risk; C: Funnel plot between CD3 on secreting CD4 regulatory T cell and PE risk; D: Funnel plot between CD3 on activated & secreting CD4 regulatory T cell and PE risk; E: Funnel plot between CD3 on CD45RA+ CD4+ T cell and PE risk; F: Funnel plot between CD3 on CD39+ CD4+ T cell and PE risk; G: Funnel plot between CD3 on CD28+ CD45RA- CD8+ T cell and PE risk; H: Funnel plot between CD3 on CD28+ CD45RA+ CD8+ T cell and PE risk; I: Funnel plot between CD3 on CD39+ CD8+ T cell and PE risk; J: Funnel plot between CD25 on resting CD4 regulatory T cell and PE risk; K: Funnel plot between CD4 on CD39+ activated CD4 regulatory T cell and PE risk; L: Funnel plot between CD45 on HLA DR+ CD8+ T cell and PE risk.

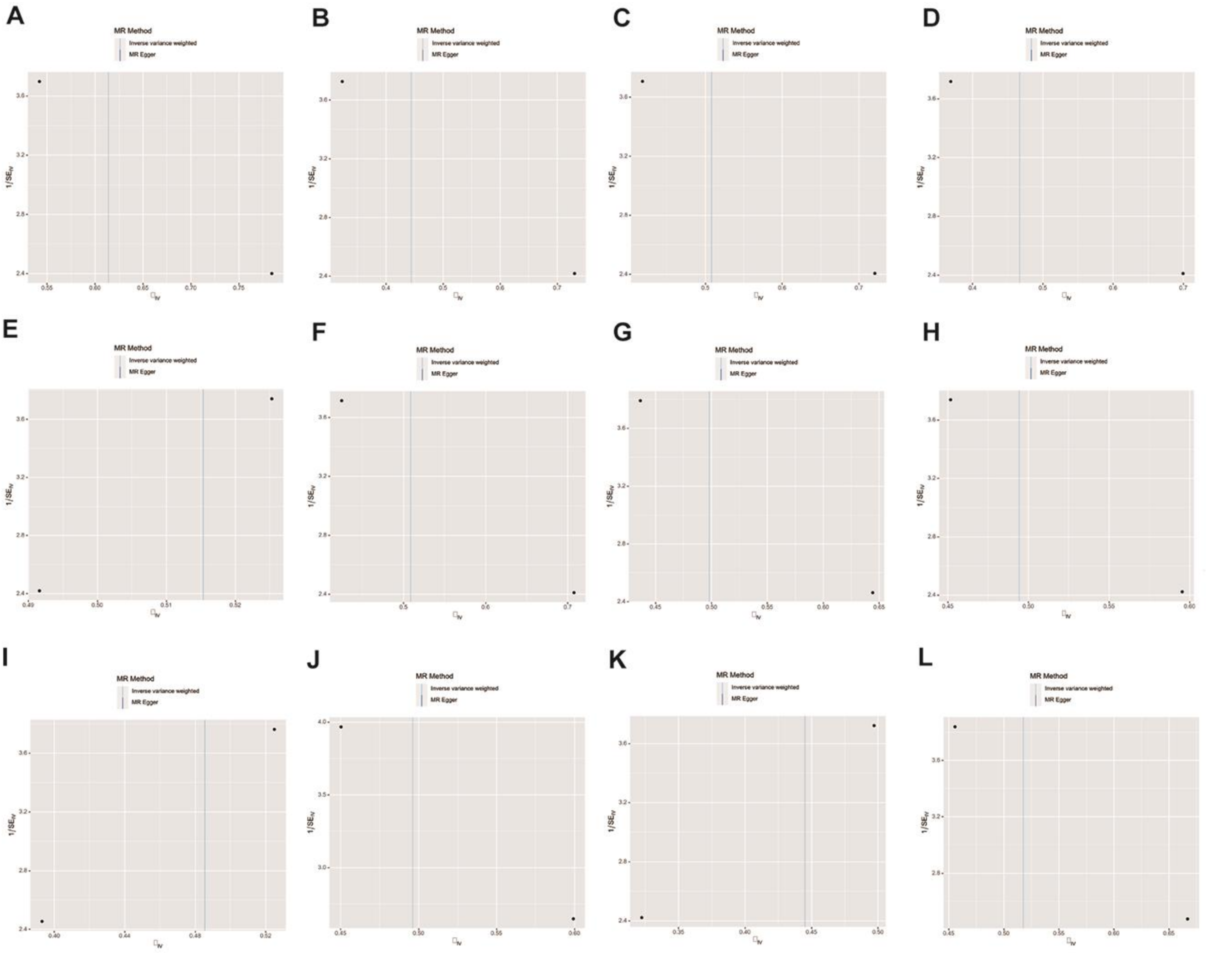

**Fig.S3 Scatter plots between PE and immune cells.** A: Scatter plot between CD3 on Effector Memory CD4+ T cell and PE risk; B: Scatter plot between CD3 on activated CD4 regulatory T cell and PE risk; C: Scatter plot between CD3 on secreting CD4 regulatory T cell and PE risk; D: Scatter plot between CD3 on activated & secreting CD4 regulatory T cell and PE risk; E: Scatter plot between CD3 on CD45RA+ CD4+ T cell and PE risk; F: Scatter plot between CD3 on CD39+ CD4+ T cell and PE risk; G: Scatter plot between CD3 on CD28+ CD45RA- CD8+ T cell and PE risk; H: Scatter plot between CD3 on CD28+ CD45RA+ CD8+ T cell and PE risk; I: Scatter plot between CD3 on CD39+ CD8+ T cell and PE risk; J: Scatter plot between CD25 on resting CD4 regulatory T cell and PE risk; K: Scatter plot between CD4 on CD39+ activated CD4 regulatory T cell and PE risk; L: Scatter plot between CD45 on HLA DR+ CD8+ T cell and PE risk.

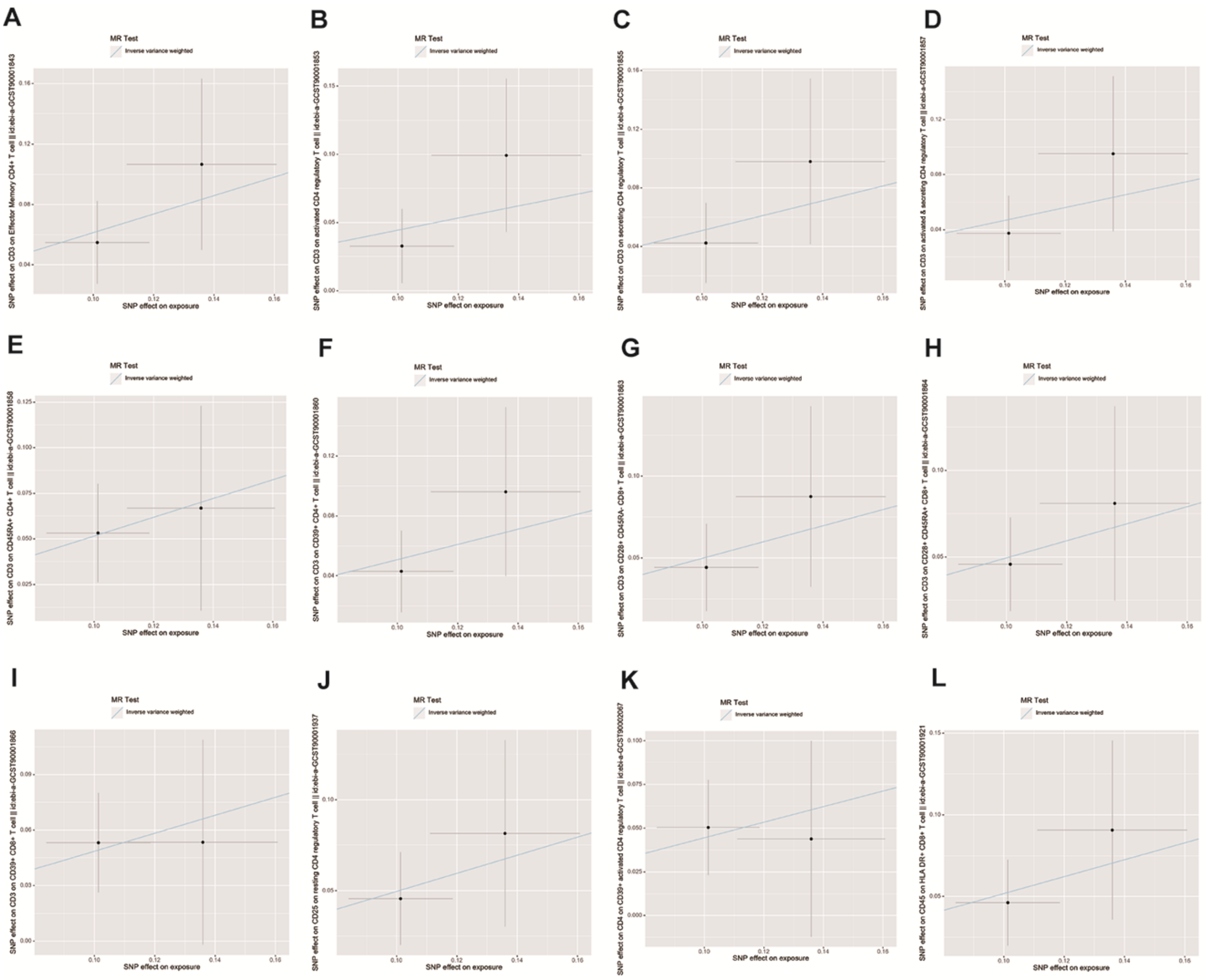

**Fig.S4 Forest plots between PE and immune cells.** A: Forest plot between CD3 on Effector Memory CD4+ T cell and PE risk; B: Forest plot between CD3 on activated CD4 regulatory T cell and PE risk; C: Forest plot between CD3 on secreting CD4 regulatory T cell and PE risk; D: Forest plot between CD3 on activated & secreting CD4 regulatory T cell and PE risk; E: Forest plot between CD3 on CD45RA+ CD4+ T cell and PE risk; F: Forest plot between CD3 on CD39+ CD4+ T cell and PE risk; G: Forest plot between CD3 on CD28+ CD45RA- CD8+ T cell and PE risk; H: Forest plot between CD3 on CD28+ CD45RA+ CD8+ T cell and PE risk; I: Forest plot between CD3 on CD39+ CD8+ T cell and PE risk; J: Forest plot between CD25 on resting CD4 regulatory T cell and PE risk; K: Forest plot between CD4 on CD39+ activated CD4 regulatory T cell and PE risk; L: Forest plot between CD45 on HLA DR+ CD8+ T cell and PE risk.

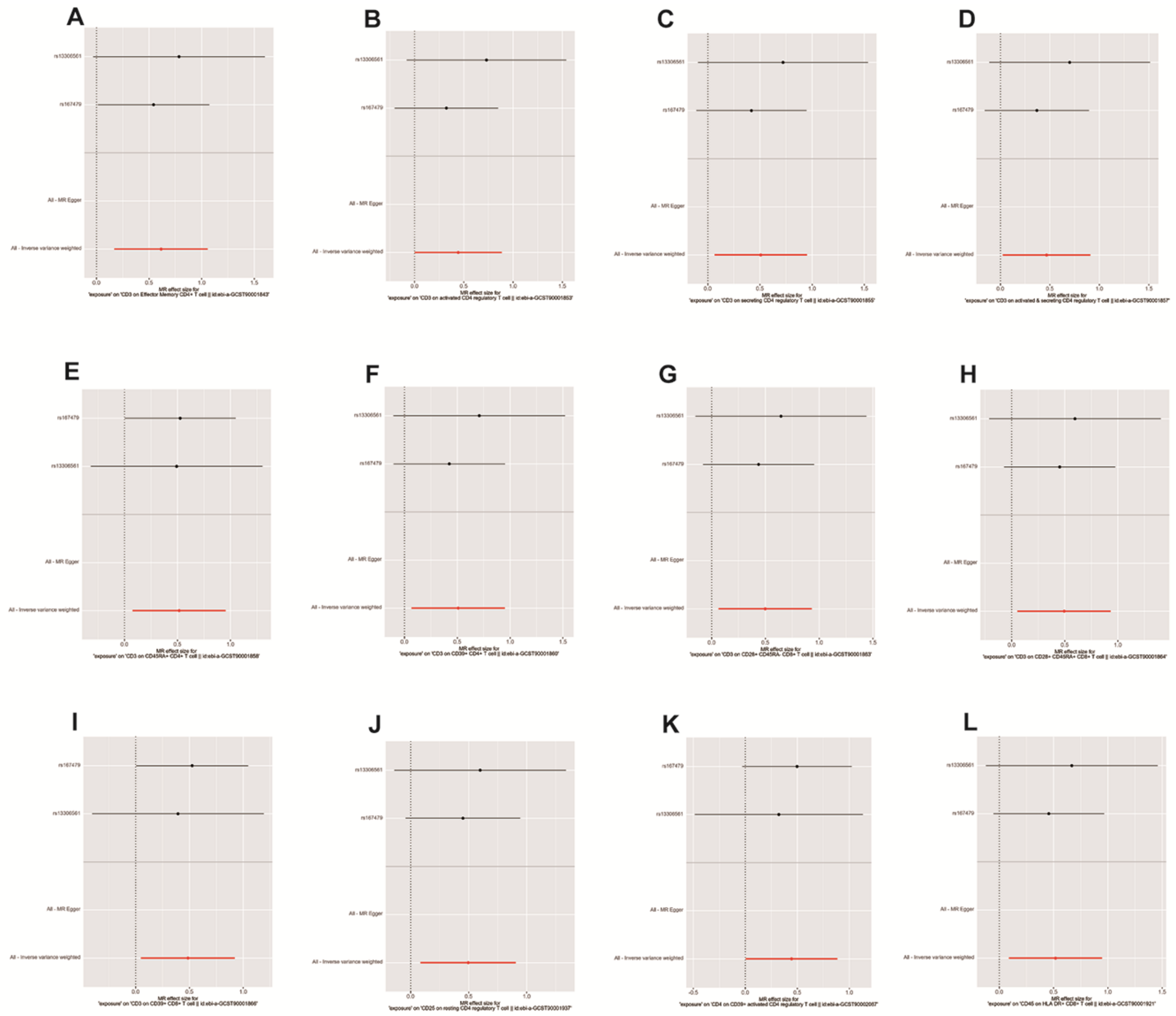

Supplement: Supplementary file 2 [file medi-105-e46002-s002.pdf]
